# Recommendations from a European Roundtable Meeting on Best Practice Healthy Infant Skin Care

**DOI:** 10.1111/pde.12819

**Published:** 2016-02-26

**Authors:** Ulrike Blume‐Peytavi, Tina Lavender, Dorota Jenerowicz, Irina Ryumina, Jean‐Francois Stalder, Antonio Torrelo, Michael J. Cork

**Affiliations:** ^1^ Department of Dermatology and AllergyClinical Research Center for Hair and Skin ScienceCharité‐Universitätsmedizin BerlinBerlinGermany; ^2^ School of Nursing, Midwifery and Social WorkUniversity of ManchesterManchesterUK; ^3^ Department of DermatologyPoznan University of Medical SciencesPoznanPoland; ^4^ Research Center for Obstetrics, Gynecology and PerinatologyMinistry of Healthcare of RussiaFederal State Budget InstitutionMoscowRussia; ^5^ Department of DermatologyNantes University HospitalNantesFrance; ^6^ Department of DermatologyHospital Infantil Universitario Niño JesúsMadridSpain; ^7^ Academic Unit of Dermatology Research, Department of Infection and ImmunityFaculty of Medicine, Dentistry and HealthUniversity of Sheffield Medical SchoolSheffieldUK

## Abstract

**Background:**

European roundtable meeting recommendations on bathing and cleansing of infants were published in 2009; a second meeting was held to update and expand these recommendations in light of new evidence and the continued need to address uncertainty surrounding this aspect of routine care.

**Methods:**

The previous roundtable recommendations concerning infant cleansing, bathing, and use of liquid cleansers were critically reviewed and updated and the quality of evidence was evaluated using the Grading of Recommendation Assessment, Development and Evaluation system. New recommendations were developed to provide guidance on diaper care and the use of emollients. A series of recommendations was formulated to characterize the attributes of ideal liquid cleansers, wipes, and emollients.

**Results:**

Newborn bathing can be performed without harming the infant, provided basic safety procedures are followed. Water alone or appropriately designed liquid cleansers can be used during bathing without impairing the skin maturation process. The diaper area should be kept clean and dry; from birth, the diaper area may be gently cleansed with cotton balls/squares and water or by using appropriately designed wipes. Appropriately formulated emollients can be used to maintain and enhance skin barrier function. Appropriately formulated baby oils can be applied for physiologic (transitory) skin dryness and in small quantities to the bath. Baby products that are left on should be formulated to buffer and maintain babies’ skin surface at approximately pH 5.5, and the formulations and their constituent ingredients should have undergone an extensive program of safety testing. Formulations should be effectively preserved; products containing harsh surfactants, such as sodium lauryl sulfate, should be avoided.

**Conclusion:**

Health care professionals can use these recommendations as the basis of their advice to parents.

Cleansing is fundamental to the care of all babies; there is growing realization that suboptimal routine skin care may alter the balance of genetic and environmental factors to transform the healthy epidermis into one associated with barrier dysfunction and attendant disease, such as atopic dermatitis (AD) 1. Therefore there is a need for evidence‐based recommendations to guide health care professionals and parents in this critical aspect of care. This is particularly pertinent in view of confusion that parents and health care workers express regarding the use of water alone for bathing versus the use of bathing products 2.

The recommendations of the first European roundtable meeting were published in 2009 3; a second meeting was convened to review and update these recommendations in light of new data and to expand their scope to include diaper care and the application of emollients and oils to healthy skin and to assess the strength of recommendations using an established grading system.

## Methods

An expert panel, all of whose members have expertise in infant skin care, reviewed the 2009 recommendations 3. A comprehensive review of published literature was conducted before the meeting to identify articles published since the previous meeting that might inform the recommendations update.

The panel evaluated the recommendations to determine whether to retain, revise, or remove each. The evidence base for each recommendation was assessed and the strength of each recommendation was assigned using the Grading of Recommendations Assessment, Development and Evaluation (GRADE) system (Table 1) 4. Consensus on the wording of each recommendation was required to be unanimous; consensus on the proposed rating of evidence or recommendation was reached when the majority (>7) was in favor.

**Table 1:** Grading of Recommendations Assessment, Development and Evaluation System of Evaluating Quality of Evidence 4

Grade	
Evidence	
High quality	Further research is very unlikely to change confidence in the estimate of effect
Moderate quality	Further research is likely to have an important effect on confidence in the estimate of effect and may change the estimate
Low quality	Further research is very likely to have an important effect on confidence in the estimate of effect and is likely to change the estimate
Very low quality	Any estimate of effect is very uncertain
Recommendation	
Strong	Desirable effects of an intervention clearly outweigh the undesirable effects or clearly do not
Weak	Low quality of evidence or the evidence suggests that desirable and undesirable effects are closely balanced

Recommendations were formulated for healthy, full‐term babies, although evidence relating to preterm infants was discussed if it was thought to be relevant to full‐term babies.

### First Cleansing

The previous recommendations 3 were modified in light of expert opinion (Table 2) rather than new direct evidence; all first‐cleansing recommendations were regarded to be founded on low‐quality evidence, a consequence in part of the considerable ethical difficulties inherent in conducting controlled studies in this group.

**Table 2:** Recommendations on the First Cleansing of (Full‐Term) Newborns

Original recommendation (Blume‐Peytavi et al 3)	Revised recommendation	Evidence strength	Recommendation strength
Immediately after delivery, baby can be wiped with water	**Baby can be wiped immediately after birth, preferably with dry towel**	Low quality	Strong
Timing of first bath should be according to local culture	Timing of first bath should be according to local culture	Low quality	Weak
Newborn's temperature should be stabilized before first bath is given	First bath should be conducted only once newborn's temperature has stabilized	Low quality	Strong
Health care workers should use gloves for first bath	Health care workers should **ideally** use gloves for first bath	Low quality	Strong

Bold indicates changes between the original and new recommendations.

The use of water for the first wiping of infants was removed from the first recommendation because this does not reflect usual practice and may be counterproductive; one of the principal reasons for wiping is to dry the baby for temperature stabilization. Furthermore retaining the vernix caseosa in the immediate postbirth period was thought to offer benefits in terms of skin protection; it is thought that the vernix caseosa provides physical protection from amniotic fluid and enzymes and lowers skin surface pH, provides lipids, and exerts a moisturizing effect 5. Therefore the first recommendation was reworded to state that a dry towel should preferably be used. Vigorous rubbing of the baby should be avoided, and in cases of significant soiling, water can be used.

That the first bath should be conducted only once the newborn's temperature has stabilized was a strong recommendation, Bathing “too early” remains commonplace in some locations 6 and should be discouraged because it may unnecessarily interrupt breastfeeding and skin‐to‐skin contact 6 and increase the risk of hypothermia and respiratory distress 7, 8.

The final recommendation was altered to state that health care workers should ideally use gloves for the first bath. This strong guidance is predicated on the assumption that maternal blood may pose a threat to health care professionals and that health care professionals not wearing gloves may increase the risk of microbial contamination to babies. Gloves were regarded as ideal but not mandatory because the first bath may occur many days after birth, at which point the risk of contamination will have decreased; there is also a limited supply of gloves in some health care settings.

### Routine Bathing

Newborns can be bathed without harm 7, 8, 9, 10, 11, 12; this assertion has gained further strength from the results of a randomized controlled study comparing a bath with use of a washcloth 13 (Table 3). Bathing—as part of an evening routine—may be advantageous in terms of improving infant and toddler sleep and maternal mood 14. This, along with the opportunity provided by bathing for enjoyment, tactile stimulation and infant–caregiver bonding 8, 11, is an aspect of routine health care that professionals may wish to consider when advising parents. Nevertheless, the panel did not think that the psychological benefits of bathing noted in the previous roundtable meeting 3 warranted a specific recommendation.

**Table 3:** Recommendations on Routine Bathing of Newborns and Infants

Original recommendation (Blume‐Peytavi et al 3)	Revised recommendation	Evidence strength	Recommendation strength
Bathing does not harm the baby	Bathing of newborns can be carried out with no harm to baby	Moderate quality	Strong
Routine bathing may begin before umbilical cord has fallen off, but there may be advantages associated with waiting	Bathing may begin before umbilical cord has fallen off	Low quality	Weak
Bathing is better than washing with a cloth	Bathing is **preferable** to washing with a cloth	Moderate quality	Strong
Bathing in the evening can help to calm baby and improve sleep	As part of an evening routine, bathing may be considered to help improve sleep	Low quality	Weak
For newborns, bathing should last 5–10 minutes	In newborns, bathing should last 5–10 minutes	Low quality	Weak
Bathing should be conducted two to three times per week until baby is crawling or as often as local culture requires	Bathing should be conducted **at least** two to three times a week or as often as local culture requires	Low quality	Strong

Bold indicates changes in content between the original and new recommendations.

A number of studies have shown that bathing before the umbilical cord has fallen off does not cause harm 10, 11 and that bathing is no worse than using alcohol wipes to cleanse the cord area 15. The clause concerning possible advantages associated with waiting was removed because of a lack of empirical evidence in this regard.

The recommendation that bathing is “better” than washing with a cloth was reworded to state that this manner of bathing is “preferable.” Although there is evidence that skin barrier parameters are better with bathing than with cloth or sponge washing 13, such differences may not be clinically significant.

The sleep recommendation was clarified in light of clinical evidence suggesting that bathing, as part of a specific routine, may improve infant sleep 14. Results of studies in adults support the argument that prebedtime bathing may be soporific through mechanisms involving the temporary modification of core temperature 16, 17.

The recommendation that newborns should be bathed for 5 to 10 minutes was retained. To our knowledge, no studies have evaluated the length of time newborns should be bathed, so the recommendation was regarded as weak, based on low‐quality evidence. Between 5 and 10 minutes reflects the duration of bathing in clinical trials, none of which suggested evidence of harm caused by the practice 11, 13, 18, 19, 20. This recommendation was limited to newborns, because older infants may enjoy longer baths, and there is no evidence to suggest that this is likely to be harmful.

The frequency‐of‐bathing recommendation was reworded to state that infants should be bathed at least two to three times per week, because this is the minimum frequency used in clinical studies 16, 21, 22, 23, 24.

### Safety While Bathing

There were few changes to the 2009 recommendations regarding safety while bathing (Table 4). It was agreed that the bath and bath toys should be “kept clean” rather than “disinfected,” acknowledging that home bathing does not warrant a disinfectant protocol. The bath and toys should be kept clean since outbreaks of Pseudomonas aeruginosa have been linked to contaminated baths and toys 21, 25. In the hospital setting, disinfecting equipment is recommended to avoid nosocomial infection 21. The room air temperature recommendation was revised to include temperatures up to 24°C, based on the assertion that in many parts of the world, such a temperature would be regarded as “comfortable” and would not expose the infant to overheating.

**Table 4:** Recommendations on Safety While Bathing Infants

Original recommendation (Blume‐Peytavi et al 3)	Revised recommendation	Evidence strength	Recommendation strength
Bath should be placed in a safe place	Bath should be located in a safe place	Low quality	Strong
Bath and any bath toys should be disinfected to avoid microbiological contamination	Microbiological contamination should be avoided by **keeping bath and bath toys clean**	Moderate quality	Strong
Water temperature should be 37°C–37.5°C	Water temperature should be 37°C–37.5°C	Low quality	Strong
Water depth should be to infant's hips	Depth of water should be to the infant's hips (**∼5 cm**)	Low quality	Weak
Washcloth may be used to cover or splash water onto the belly to maintain body heat	Washcloth may be used to cover or splash water onto belly to maintain body heat	Low quality	Strong
Room air temperature should be 21°C–22°C	Room air temperature should be 21°C–**24**°C	Low quality	Weak
Baby should not be left alone while in bath and young children should not be allowed to wash the baby	Baby should not be left alone in the bath and young children should not be allowed to wash the baby	Low quality	Strong
If oils are used, a mat should be placed in the bath which should also be disinfected regularly	If oils are used, a mat should be placed in the bath, which should also be disinfected regularly	Low quality	Strong

Bold indicates changes between the original and new recommendations.

### After Bathing

Recommendations regarding postbathing care were strengthened slightly (Table 5). It was recommended that infants be covered—not specifically clothed—after bathing. Covering will help prevent the significant temperature drop that can occur 10 minutes after neonatal bathing 12, but unlike clothing, it does not preclude the opportunity for skin‐to‐skin contact with the parent. Two strong recommendations were developed to distinguish between the management of dry skin that results from “normal” maturation processes, such as neonatal physiologic desquamation 22, 26, 27, and conditions that might warrant prescription medication, such as AD. There is clinical evidence to support the efficacy of emollients in improving skin barrier function 23, 24 and preventing diaper dermatitis 28.

**Table 5:** Recommendations on Procedures After Bathing

Original recommendation (Blume‐Peytavi et al 3)	Revised recommendation	Evidence strength	Recommendation strength
Immediately cover the baby with a towel and pat dry	Babies should be immediately covered with a towel and patted dry	Low quality	Strong
Dress baby immediately after drying	Babies should be immediately **covered** after drying	Low quality	Strong
Changes to skin structure (e.g., dryness, fissures, flaking) should be treated with an emollient or a protective ointment (diaper area)	**An emollient or a protective ointment (diaper area) can be applied for physiological (transitory) skin dryness (see section on emollient care)**	Moderate quality	Strong
	**Any pathological damage (e.g., fissures or flaking) should be referred to an appropriate medical professional**	Low quality	Strong

Bold indicates changes between the original and new recommendations.

### The Use of Cleansers in Bathing

Surfactants are used in cleansers to remove sweat, sebum, deposits, and oils from the skin, but the interaction between cleanser surfactants and stratum corneum (SC) proteins and lipids can be damaging, potentially leading to tightening of the skin, dryness, erythema, irritation, and itch 29. Health care professionals and parents should be aware of the fundamental distinction between “soaps” and cleansers composed of synthetic detergents (syndets). Soaps are technically the salts of fatty acids (such as sodium cocoate), and soap‐based cleansers are alkaline in nature (pH 10). Bar soaps and soap‐based liquid cleansers can remove natural moisturizing factor (NMF) and lipids from the skin, potentially leading to skin irritation, erythema, and itching 29. In contrast, most of the currently available syndet‐based cleansers are pH neutral or acidic and are formulated to be significantly milder than soap (particularly if harsh surfactants such as sodium lauryl sulfate [SLS] are avoided), which results in less potential for irritation and itch 29. Syndet liquid cleansers, as opposed to syndet bars, facilitate more efficient delivery of potentially beneficial substances such as emollients and occlusives 29. Therefore syndet‐based liquid cleansers have formed the focus of recent research, and products labeled as “soaps” should be avoided on baby skin.

Liquid cleansers that are appropriately formulated for babies are well tolerated and noninferior to washing with water alone 18, 19, 20, 30. It has also been shown that such cleansers do not alter the natural process of maturing skin barrier function in healthy full‐term newborns 19. In addition to being soap free (syndet based), a defining characteristic of the liquid cleansers used in these studies is that they are of neutral or slightly acidic pH, a characteristic echoed in the recommendations for neonatal skin care published by the Association of Women's Health, Obstetric and Neonatal Nurses 24. Therefore the panel strongly recommended that baby skin may be cleansed with water alone or by adding an appropriately formulated liquid cleanser to water and that appropriately designed liquid cleansers can be used without impairing the skin maturation process. In line with the manner in which babies were cleansed in relevant studies 18, 19, 20, 30, these two recommendations apply to the whole body and do not exclude the diaper area (Table 6). The recommendations do not stipulate that liquid cleansers “should” be used, but that they “can” be used. The cost and availability of liquid cleansers may preclude their appropriate use in some settings; nevertheless, parents who intend to use such products should be reassured that their use does not harm normal skin maturation processes.

**Table 6:** New Recommendations on the Use of Liquid Cleansers for Bathing

New recommendation	Evidence strength	Recommendation strength
Baby skin may be cleansed with water alone or by adding an appropriately formulated liquid cleanser^a^ to water	Moderate quality	Strong
Appropriately designed liquid cleansers can be used without impairing the skin maturation process	Moderate quality	Strong
Parents and caregivers must read product instructions and abide by them; labels should be clear and easy to understand	Low quality	Strong
Products supported by robust clinical data should be selected over those that are not similarly developed	Low quality	Strong

a Liquid cleansers that are free of known irritants and neutral or mildly acidic (pH 5.5–7.0) or have minimal effect on the baby's skin surface pH 24.

It was acknowledged that parity in terms of safety and effectiveness does not exist between liquid cleansers and that products, unlike water, may cause harm if misused. Therefore it was recommended that products associated with robust clinical data should be selected over those that are not (Table 6).

### Diaper Care

Diaper dermatitis is common, affecting most infants at least once 31. The occlusive diaper environment plays host to a complex interplay of potentially damaging factors. Alkaline urine disrupts the pH balance of the epidermis, which encourages microbial overgrowth and activation of fecal lipases, endogenous and exogenous proteases and bile salts, all of which can lead to further injury 32, 33. New recommendations were developed to provide guidance on routine care of the diaper area (Table 7).

**Table 7:** New Recommendations on Diaper Care

New recommendation	Evidence strength	Recommendation strength
Diaper area should be kept clean and dry	High quality	Strong
Diapers should be changed as often as necessary to ensure that skin is kept clean and dry	Moderate quality	Strong
Skin of the diaper area should be gently cleansed with cotton balls/squares or washcloth and water alone or using specifically designed wipes	Moderate quality	Strong
Appropriately designed wipes^a^ can be used from birth	Moderate quality	Strong
Wipes can be used at each diaper change	Moderate quality	Weak
Drying can be achieved through air drying or gentle patting with a dry towel or dry cotton balls/squares	Moderate quality	Strong
Diaper area should be cleaned before bathing, if necessary	Moderate quality	Strong

a Wipes should contain pH buffers to maintain slight acidity of the skin and should be free of potential irritants such as alcohol, fragrance, essential oils, soap, and harsh detergents (e.g., sodium lauryl sulfate); they should contain well‐tolerated preservatives.

To reduce the risk of diaper dermatitis and manage the condition should it occur, it was strongly recommended that the diaper area be kept clean and dry and that diapers should be changed as often as necessary. The need to keep the diaper area dry was highlighted, as prolonged and excessive humidity can increase friction, lead to skin maceration, increase permeability, and encourage microbial overgrowth 34. Because excessive scrubbing and rubbing of the diaper area may promote irritation and further damage the barrier properties of the skin surface 24, 35, 36, it was strongly recommended that drying should be achieved through air drying or gentle patting with a dry towel or dry cotton balls/squares.

A number of studies have directly compared the use of wipes with water 37, 38, 39, 40, 41, 42, 43, 44. None were powered to assess the superiority of one form of cleansing over the other, although there was no evidence from any study to indicate that wipes caused harm 37, 38, 39, 40, 41, 42, 43, 44. Based on a review of these studies, it was recommended that the diaper area be gently cleansed with cotton balls/squares or a washcloth and water alone or by using appropriately designed wipes (strong recommendation) (Table 7). Wipes appropriately designed for infants may be used from birth and at each diaper change. As the authors of a randomized controlled equivalence study comparing wipes with water 41 and others 43 found, one of the most important characteristics of baby wipes that makes them specifically suitable for babies is pH; wipes should contain pH buffers to maintain the slight acidity of skin. Other considerations are that they should be free of potential irritants such as alcohol, fragrance, essential oils, soap, and harsh detergents (e.g., SLS) 41. Given that wet wipes provide the ideal environment for microbial growth 45, it is important that wipes contain appropriate (well‐tolerated) preservatives, as regulated by bodies such as the U.S. Cosmetic Ingredient Review or the European Union Cosmetics Directive. Health care professionals should be aware of recent reports of allergic contact dermatitis in children in relation to methylisothiazolinone used in wet wipes 46, 47. Water used for wiping with cotton balls/squares or a washcloth does not need to be boiled unless there is a specific concern about the water quality. Given reports of neonatal deaths in the intensive care unit attributable to infected bathing water in developing countries 48, this recommendation does not extend to low‐birthweight or premature babies.

### Use of Emollients

Clinical evidence derived from controlled studies of healthy infants 19, 49, 50 combined with evidence from studies demonstrating the positive effect of emollients in patients with AD (or at high risk of AD) and impaired barrier function 51, 52, 53, 54 supports the theoretical rationale for the use of emollients 55. Appropriately formulated emollients can be used to maintain or enhance skin barrier function after bathing and at least twice‐weekly application of emollients, as in the Garcia Bartels et al study 19, should be considered (Table 8). Longer‐term studies designed to evaluate the clinical benefits of emollients in healthy infants—potentially to prevent the onset of the typical progression of allergic diseases that often begins early in life—are warranted 56 and would strengthen the evidence for these recommendations.

**Table 8:** New Recommendations on Topical Use of Emollients and Oils

New recommendation	Evidence strength	Recommendation strength
Recommendations for use of emollients		
Appropriately formulated emollients can be used to maintain or enhance skin barrier function	Low quality	Strong
Emollients can be used after bathing	Low quality	Strong
At least twice‐weekly application of emollients should be considered	Low quality	Strong
Choice of emollient should be adapted to geographical location and seasonal variations	Low quality	Strong
Emollients should be applied as a thin layer, with special attention to certain areas	Low quality	Strong
Recommendations for topical application of oils		
Appropriately formulated baby oils can be lightly applied to skin for physiological (transitory) skin dryness	Low quality	Weak
A small amount of appropriately formulated baby oil can be applied to baby's bath, according to product usage recommendations	Low quality	Weak
Over‐the‐counter cooking or kitchen oils should not be used on baby's skin	Low quality	Strong

Given that environmental factors such as humidity and temperature can affect the skin, it was strongly recommended that the choice of emollient be adapted to geographic location and season 57, 58. Richer (higher proportion of emollient than water) emollients may be required in cold climates and less rich emollients in warmer climates or times of the year.

Emollients should be applied in a thin layer to avoid occlusive effects, and care should be taken to avoid the trapping of emollients in folds, which may lead to dysregulation of evaporation or heat dysregulation and bacterial colonization 59, 60. Special attention should be paid to the perioral area to protect infants from the irritating effects of saliva, particularly during periods of teething. If neonatal or infantile acne is present, application of emollients to the affected area should be avoided to prevent follicular occlusion, which could exacerbate the condition.

Highlighting the importance of design, certain emollients containing SLS and no humectants may contribute to skin barrier breakdown (SLS solubilizes SC lipids, denatures keratin, and raises skin surface pH 32), so health care professionals should be aware of the suitability of emollients, and those containing SLS should be avoided.

### Use of Oils

Oils are widely used as emollients during bathing and to lubricate the skin during infant massage. Research suggests that infant massage, particularly when a lubricant is used, may offer several positive effects, including improving neonatal jaundice and weight gain 61, 62, 63, but no controlled studies in healthy, full‐term infants have evaluated the effect of the direct application of oils or the addition of oils to bath water on normal skin.

In many parts of the world there is a long history of using locally produced oils on infant skin, but vegetable oils vary greatly in terms of chemical composition. Many cookery and kitchen vegetable oils are sensitive to oxidation or light and are associated with variable levels of biologic activity 64. Therefore the effects of topically applied grocery vegetable oils are unpredictable because vegetable oils are chemically heterogeneous, individual bottles of the same type of oil may differ chemically because of the natural variability in source ingredients, and the characteristics of an individual bottle of oil will likely change over time as oxidative processes take place. Pharmaceutical‐grade oils, including mineral oil, are chemically inert, stable, and generally regarded as safe, exerting a moisturizing effect by hydrating and enhancing the SC 64. As such, appropriately formulated baby oils can be applied in a thin layer to the skin for physiological (transitory) skin dryness, and a small amount of appropriately formulated baby oil can be applied to the bath (as long as appropriate safety measures are in place [see Table 4]; it is particularly important that infants not be left alone with a bottle of oil and that products be used as directed to reduce the risk of infants breathing in or ingesting oils) (Table 8).

Research is ongoing regarding the safety–efficacy profile of specific vegetable and seed oils. The use of vegetable oils in specifically designed products (rather than the direct application of natural oils on the skin) is a separate question beyond the scope of this article.

### Ideal Cleansers, Wipes, and Emollients

The results of the studies of products described above cannot necessarily be extrapolated to all similarly described products. To help guide the manufacturers of baby skin care products, “ideal” product attributes have been recommended (Table 9). It is hoped that these recommendations will serve as a benchmark for the future development of cleansers, wipes, and emollients. Translation of these recommendations into practical guidance to aid product recommendation and selection could warrant the development of a system by which individual products are rated for suitability for infant skin according to a set of predefined criteria. Such a system, if clear and widely adopted, has the potential to simplify choice and help protect infants from suboptimal products.

**Table 9:** New Recommendations for Industry on Properties of Ideal Liquid Cleansers, Wipes, and Emollients

New recommendation	Evidence strength	Recommendation strength
Formulation should buffer or maintain babies’ skin surface at approximately pH 5.5	Moderate quality	Strong
Formulation should not interfere with normal skin microbiome development of babies’ skin	Low quality	Strong
Formulation and all of its ingredients should have undergone extensive program of safety testing; only use ingredients approved for use on babies by regulators^a^	Low quality	Strong
Formulation should be effectively preserved using preservatives recommended by regulators^a^	Low quality	Strong
If formulation contains fragrances, they should be selected from a regulator‐approved list, which should have the lowest probability of causing adverse events, such as contact dermatitis^a^	Low quality	Strong
Safety and efficacy of the formulation should be evaluated in high‐quality clinical trials	Low quality	Strong
Formulation should contain a complex of mild emulsifiers and surfactants that will effectively cleanse or hydrate babies’ skin and exert no negative effects on skin barrier	Low quality	Strong
Cleanser may contain emollient ingredients, and emollient should contain a combination of ingredients that will have a positive effect on skin barrier	Low quality	Strong
Formulation for liquid cleanser and wipes should effectively cleanse babies’ skin to remove substances that may be damaging to it, such as feces, urine, and food residues	Low quality	Strong
Formulation should not contain ingredients that will damage babies’ skin, such as harsh surfactants, in particular sodium lauryl sulphate	Moderate quality	Strong
Formulation should not irritate babies’ skin (cleanser, wipes, emollients) or eyes (cleanser only)	Moderate quality	Strong

a There is no comprehensive and universal list of ingredients approved for use on baby skin, but the U.S. Food and Drug Administration has published a list of banned substances for use in cosmetic products 65. The European Commission has published a list of approved preservatives for use in cosmetics 66, and information about approved fragrances can also be searched for 67.

## Conclusion

Skin care is fundamental to healthy infant development, but parents may be confused regarding best practice for this routine aspect of care 2. We hope that health care professionals will use these evidence‐based recommendations to guide parents with respect to the practical facets of cleansing, bathing, moisturizing, and care of the diaper region. Critical updates from the previous recommendations have been derived from clinical studies of liquid cleansers, wipes, and emollients; for example, the equivalence of wipes 41 and the noninferiority of a liquid cleanser 18 to water have been shown (in terms of skin hydration and transepidermal water loss, respectively), and studies have demonstrated that the use of such products does not influence skin barrier maturation 19, 37. Therefore clinical evidence demands a nuanced approach; parents should be reassured in their choice of infant skin care regime, be it the sole use of water or the use of appropriately formulated cleansers, wipes, emollients, and oils.

## Conflict of Interest

UB‐P is a consultant to Johnson & Johnson, has received honoraria as a speaker for Pierre Fabre and Galderma, and has received grants for clinical and research trials from Galderma, Johnson & Johnson, Pierre Fabre, Bübchen, and WALA as an employee of the Charité. MJC and TL have received research grants from, given lectures for, and acted as consultants to Johnson & Johnson. DJ is a substantive consultant for Polish Johnson's Baby publications and materials. AT has been paid as a consultant for Johnson & Johnson trials.

## Financial Support

The roundtable meeting was supported by Johnson & Johnson, but the company did not contribute to, review, or author this article. Medical writing assistance for the development of this article was provided by Amy Jackson, M.Phil., at Fishawack Communications, whose services Johnson & Johnson paid for.
